# An Overview of CMOS Photodetectors Utilizing Current-Assistance for Swift and Efficient Photo-Carrier Detection

**DOI:** 10.3390/s21134576

**Published:** 2021-07-04

**Authors:** Gobinath Jegannathan, Volodymyr Seliuchenko, Thomas Van den Dries, Thomas Lapauw, Sven Boulanger, Hans Ingelberts, Maarten Kuijk

**Affiliations:** 1 Department of Electronics and Informatics (ETRO), Vrije Universiteit Brussel, 1050 Brussels, Belgium; vse@melexis.com (V.S.); Thomas.Van.Den.Dries@vub.be (T.V.d.D.); Thomas.Lapauw@vub.be (T.L.); sven.boulanger@vub.be (S.B.); hans.ingelberts@vub.be (H.I.); mkuijk@vub.be (M.K.); 2 Melexis, Transportstraat 1, 3980 Tessenderlo, Belgium

**Keywords:** current-assistance, CAPD, CMOS, fast time-gated cameras, demodulation, avalanche, Geiger

## Abstract

This review paper presents an assortment of research on a family of photodetectors which use the same base mechanism, current assistance, for the operation. Current assistance is used to create a drift field in the semiconductor, more specifically silicon, in order to improve the bandwidth and the quantum efficiency. Based on the detector and application, the drift field can be static or modulated. Applications include 3D imaging (both direct and indirect time-of-flight), optical receivers and fluorescence lifetime imaging. This work discusses the current-assistance principle, the various photodetectors using this principle and a comparison is made with other state-of-the-art photodetectors used for the same application.

## 1. Introduction

Over the years, the prefix “current-assistance” has been found in an increasing number of different photonic sensors: current-assisted photonic demodulators (CAPD) for time-of-flight imaging [1,2,3,4,5,6,7,8,9,10,11,12,13,14,15,16,17,18], fast time-gated current-assisted photonic samplers (CAPS) for high-precision fluorescence lifetime imaging [19,20,21,22], current-assisted photodetector (CAP) for optical receivers [23] and current-assisted single-photon avalanche diodes (CA-SPAD) for fast and efficient single-photon detection [24,25]. This review paper focusses on research performed at the Department of electronics and informatics (ETRO), Vrije Universiteit Brussel (VUB) using this “current-assistance” principle.

## 2. Current-Assistance Principle

**Figure 1:**
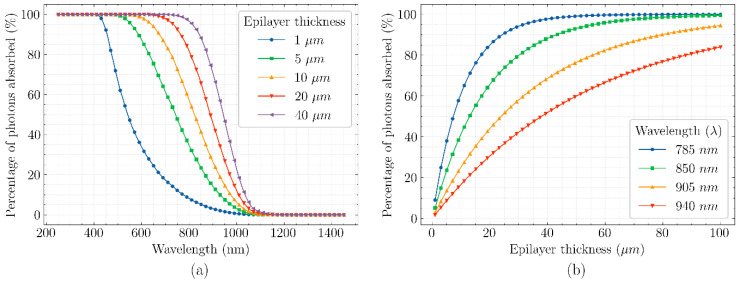
Absorption percentage in Silicon as a function of wavelength for different epilayer thicknesses (**a**) and absorption percentage in silicon as a function of epilayer thickness for a few wavelengths (**b**) (Absorption coefficient data from [27]).

In Silicon photodetectors, it is always desirable to have a high quantum efficiency in the spectral region of interest. For applications requiring high quantum efficiency in the near infra-red (NIR) region, a quick solution is to have a thick/deep absorption layer because more low energy (or long wavelength) photons can be absorbed. The relation between the absorption and the epilayer thickness can be seen in Figure 1a,b. Unfortunately, there are no free rides on the Quantum Efficiency Express. A large photon-absorption volume will indeed result in more photo-generated carriers, but without any electric field present in the absorption volume, the main carrier transport mechanism is diffusion. Diffusion is where the free carriers move from a region of high carrier concentration to a region of low carrier concentration [26]. Diffusion is not directional and does not always lead to free carriers ending up in the region of interest, i.e., the detection node. Moreover, diffusion is a slower process compared to drift, thus the generated photo-carriers have a higher chance of recombination leading to a loss in quantum efficiency. In short, the pixel volume is “self-limiting” because if there is no electric field in the whole volume; this leads to drop in speed and in quantum efficiency.

The other major carrier transport mechanism is drift. Due to an electric field, carriers move swiftly with a drift velocity ( $v_{d}$ ). At low electric fields, the drift velocity follows the Equation (1), where it is directly proportional to the applied electric field ( E ) and the mobility of the carriers ( μ ). Here, the movement is faster compared to diffusion and very directional. Therefore, the generated photo-carriers can be directed, with the required speed, to the region of interest where they can be detected. Usually, the drift field is fast enough that almost all generated carriers can be detected before recombining. Recombination lifetimes ( τ ) range from nanoseconds to milliseconds depending on the dopant concentration and recombination-centers concentration [28].

(1) $$v_{d}=\mu \times E$$

Traditionally, a *p-n* junction is used for photo-detection because of the built-in electric drift field that separates the generated electron-hole pairs. The electric drift field increases carrier speeds proportionally to the electric field strength (Equation (1)) up to carrier saturation velocity.

However, another way of establishing a drift field is through the application of a current through or voltage across a semiconductor material. Like with Ohm’s law, it is hard to tell whether one applies a voltage and gets a current in return or vice versa. The resulting current-assisted drift field distribution, is different from the built-in (and/or applied) drift field distribution in the case of a *p-n* junction. Since the unavoidable assisting current makes the distinction between the two origins of having a drift field, the qualifier “current assistance” has been used systematically to distinguish detectors based on this type of drift-field. The drift field due to current-assistance also follows Equation (1) up to carrier saturation velocity. 

As the origin of both types of electric field distribution are different, the possibilities, limitations, and challenges are different in each case. The current-assisted drift fields can be switched in direction, and in some cases allow more easily to fill a larger semiconductor volume with a drift field. However, the cost of applying this drift field is the current-flow between the two regions, due to a flow of majority carriers (holes in *p*-type semiconductor and electrons in *n*-type semiconductor) and its associated power dissipation.

**Figure 2:**
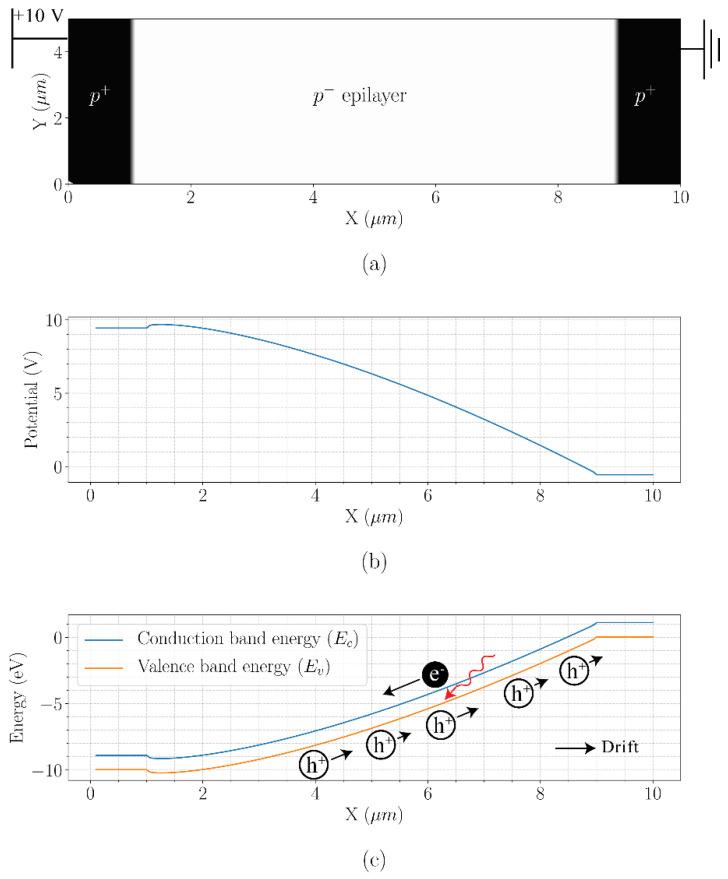
Cross-section of a simple p-i-p structure (**a**), the potential as a function of X (at any point in Y) (**b**) and the corresponding conduction and valence band energies with overlaid illustration of current-assistance operation (**c**).

For example, in a *p-i-p* semiconductor structure, when a potential difference is applied between the two *p*-type regions, there will be a flow of holes from the *p*-type region with a higher potential to the *p*-type region with a lower potential. Now, if a photon is absorbed in the *i*-region, it will generate an electron-hole pair. The photoelectron will experience a drift field towards the *p*-type region with the higher potential while the associated hole will drift in the opposite direction. This phenomenon is illustrated in Figure 2.

**Figure 3:**
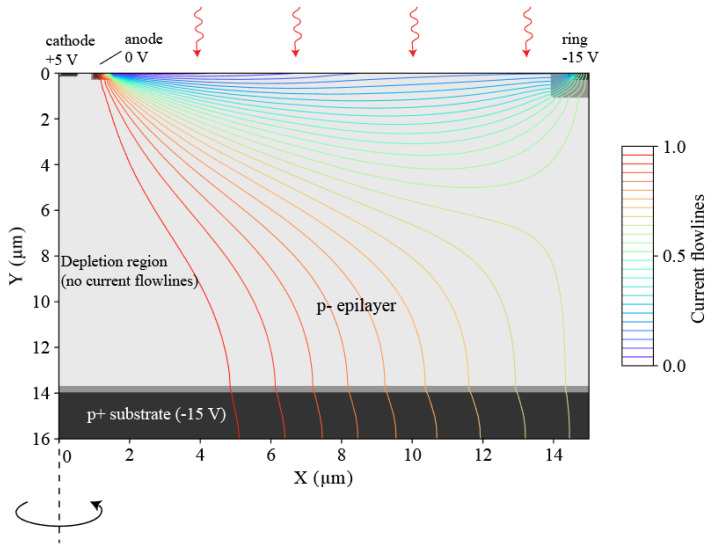
Cross section of the example structure to demonstrate current-assistance principle with an overlay of simulated current flowlines.

The current assistance principle is illustrated in Figure 3 by a planar CMOS technology compatible structure and is simulated in Silvaco Atlas device physics simulator [29]. This cylindrical photodetector, situated in a high-resistivity *p^-^* epilayer (~1000 Ω·cm), has a very small *p-n* junction diode in the center. The diode comprises of a n^+^ cathode surrounded by a p^+^ annular anode. This “central” photodiode is surrounded by a larger ring of p^+^ doping, which we will refer to as ring. The extent of the ring marks the boundaries of the photodetector and the area encapsulated by the ring is the photo-absorption area. The central *p-n* junction is reverse biased, and the ring is biased at a lower potential than the anode. Due to this potential difference, there is a hole current between the anode and the ring. The photo-generated electrons in the volume between the anode and the ring will be swiftly guided towards the anode with a drift velocity ( $v_{d}$ ). When the photo-electrons reach the anode, they only have to diffuse a very small distance to fall into the depletion region and get detected by the cathode. There is a depletion region in the epilayer, whose area is proportional to the reverse bias voltage and the doping concentration, that normally does not extend beyond the epilayer due to the high doping levels of the p^+^ substrate.

**Figure 4:**
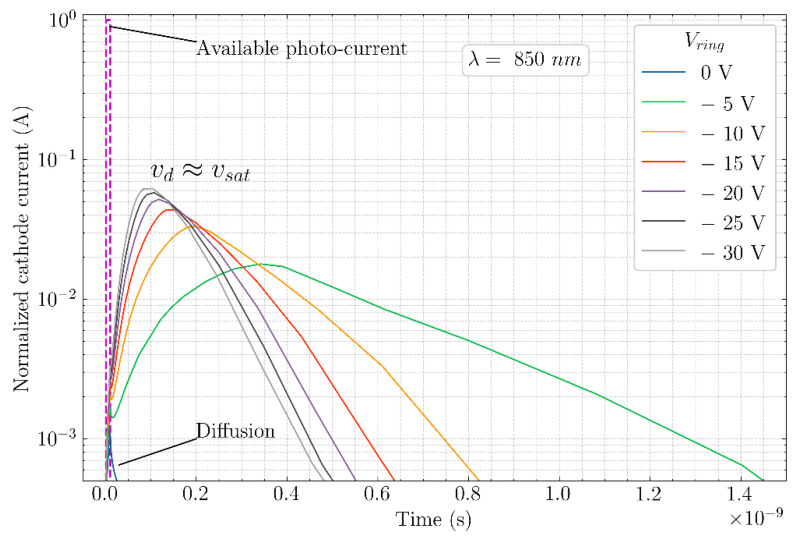
Simulated timing response of the device when illuminated with a sharp light pulse (10 ps) of 850 nm for a few different ring voltages.

Once the photoelectrons reach the vicinity of the anode region, they will have to diffuse a small distance, due to a small potential gradient around the anode, to fall into the depletion region between the anode and the cathode at which point they are detected. The photo-detection bandwidth is dictated by the size of the detector, the larger the detector the more the transit time to reach the detection area. In practice, diffusion of the photoelectrons near the anode can be a main factor which limits the bandwidth of current-assisted detectors with a lateral drift field. A simulated impulse response of the example detector, when illuminated uniformly with a sharp (10 ps), 850 nm, is shown for different ring voltages (cathode–anode voltage fixed at +5 V) in Figure 4. When the ring voltage is the same as the anode voltage, then there is no drift field and diffusion becomes the only transport mechanism, leading to only few photoelectrons reaching the cathode (the rest recombine) and thereby reducing the quantum efficiency. With increasing ring voltages (−5 V to −30 V); however, the impulse response keeps getting sharper and converges to a maximum due to velocity saturation. This structure is very similar to that of the current-assisted photodiode (CAP) which will be explained in more detail in the next section (Section 3.1).

## 3. CMOS Photodetectors Using Current-Assistance

### 3.1. Current-Assisted Photodetector (CAP)

**Figure 5:**
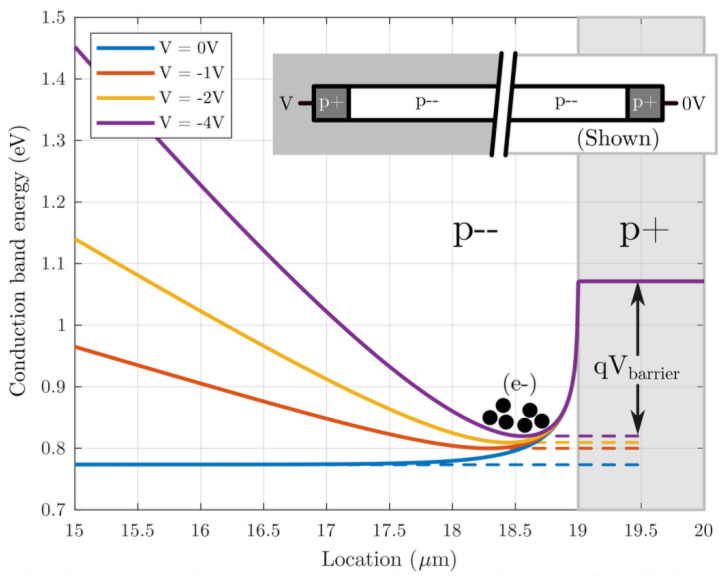
The simulated conduction band of a *p-i-p* photodetector shows how photogenerated minority charge carriers can be trapped and the cross-section of the *p-i-p* detector (inset).

At the basis of the majority current modulation lies the *p-i-p* structure, which has been shown to exhibit photogain through accumulation or trapping of minority carriers [30,31,32]. Therefore, it is recognized that the assisting majority current itself is modulated significantly by the incident light, and thus can be included to the total AC detection current. The *p-i-p* structure allows trapping of electrons inside the substrate. This helps the accumulation of holes in the substrate, which in turn reduces the overall resistivity of the detector. In general, the high–low junction barrier height, as well as the thickness of the barrier p^+^ region (see Figure 5) determines how well the electrons can be trapped. This trapping of minority carriers is known to improve responsivity dramatically, but it at the same time reduces the bandwidth just as much. This behavior is also known to be very nonlinear with respect to the incident optical power.

**Figure 6:**
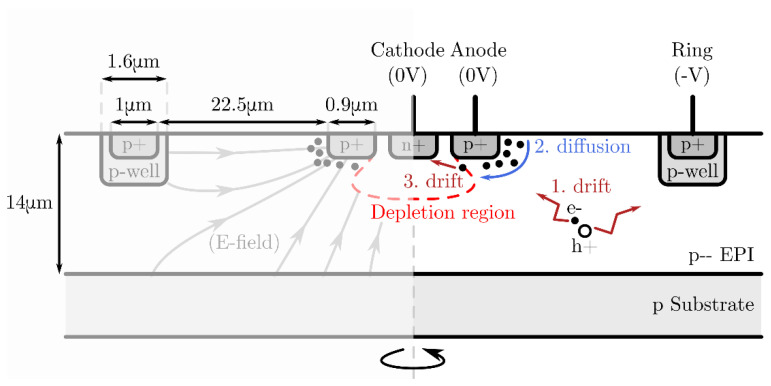
The layout and dimensions of the fabricated current-assisted photodiode. The center cathode n^+^ region is 1.5 µm in diameter, keeping 0.75 µm space with the anode p^+^ region. The ring is biased to a negative bias voltage.

The simplest photodetector that makes use of current-assistance is the Current-Assisted Photodiode (CAP) [32]. Rather than trapping all electrons inside the substrate, the CAP provides a sink for photoelectrons by means of the cathode n^+^ region. This structure is shown in Figure 6. This means that the bandwidth will be improved at the cost of photo-gain. Like with any “current-assisted” detector, we can identify three travel modes for minority carriers determining the overall bandwidth.

Initially, most minority carriers will be generated inside the substrate. At this point, a drift field is needed to bring them to the anode, often resulting in the need to apply a backside potential as well. Along the anode edge, they accumulate and diffuse into the cathode, where they can drift again once they reach the depletion region. A worst-case, average time constant for these situations can be modeled.

(2) $$\tau_{\mathrm{dr}}=\frac{l_{\mathrm{drift}}}{\mu \left|\vec{E}\right|}$$

(3) $$\tau_{\mathrm{diff}}=\frac{l_{d}^{2}}{2n_{\dim}D_{n}}$$

(4) $$\tau_{\mathrm{tot}}^{2}=\tau_{\mathrm{dr}1}^{2}+\tau_{\mathrm{diff}}^{2}+\tau_{\mathrm{dr}3}^{2}$$

Here, $l_{\mathrm{drift}}$ is the average distance traveled while in the strong drift field, while μ is the minority carrier mobility and $\left|\vec{E}\right|$ the electric field strength. $l_{d}$ is the average distance traveled along the edge of the anode p^+^-region, $n_{\dim}$ represents the number of dimensions, and $D_{n}$ is the minority-carrier diffusion constant. Usually, the combination of $\tau_{\mathrm{diff}}$ , the average time spent diffusing, and $\tau_{\mathrm{dr}1}$ , the average time spent drifting in the bulk towards the anode, decides the detection bandwidth [33]. The last drift stage (the third travel mode, $\tau_{\mathrm{dr}3}$ ) where the charge carrier is captured by the depletion region near the center cathode is often negligible. For small pixel pitches (<10 µm), $\tau_{\mathrm{diff}}$ plays a more important role in limiting bandwidth and for large pixel pitches (>100 µm) $\tau_{\mathrm{dr}1}$ can become large enough to limit bandwidth. The minority carrier transport time is dependent on the location of the photogenerated minority carriers within the device, e.g., the minority carriers that are generated directly below the cathode n^+^ region drift directly towards that n^+^ region without diffusion along the anode p^+^ region. The expected value for the total time for a minority carrier to reach the cathode (Equation (4)) is therefore a worst-case scenario.

**Figure 7:**
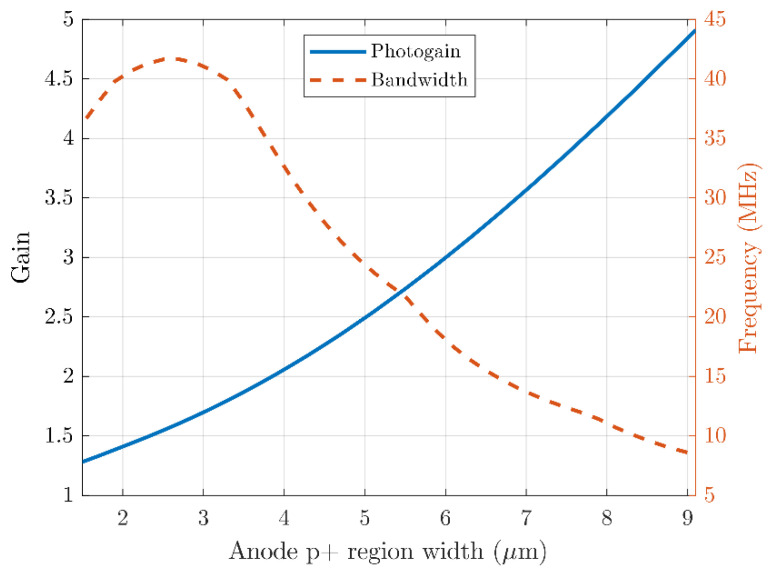
The simulated photo-gain and bandwidth for varying anode p^+^ region cross-section widths. The width can be used to trade bandwidth for photo-gain.

Figure 7 illustrates the photo-gain bandwidth tradeoff as a function of the anode region (p^+^) width. We keep $V_{cathode}=V_{anode}=0\;V$ , and have $V_{ring}=V_{substrate}=-3\;V$ . The simulated structure is cylindrically symmetric with a ring diameter of 50 µm.

For small p^+^ regions, the uniformity of the electric field inside the epitaxial layer determines the bandwidth. For larger p^+^ regions, we find that the accumulation of photogenerated electrons near the anode p^+^ region causes both an increase in photo-gain, as well as a decrease in bandwidth. For larger p^+^ regions, the DC biasing current also increases which may not be desirable.

**Figure 8:**
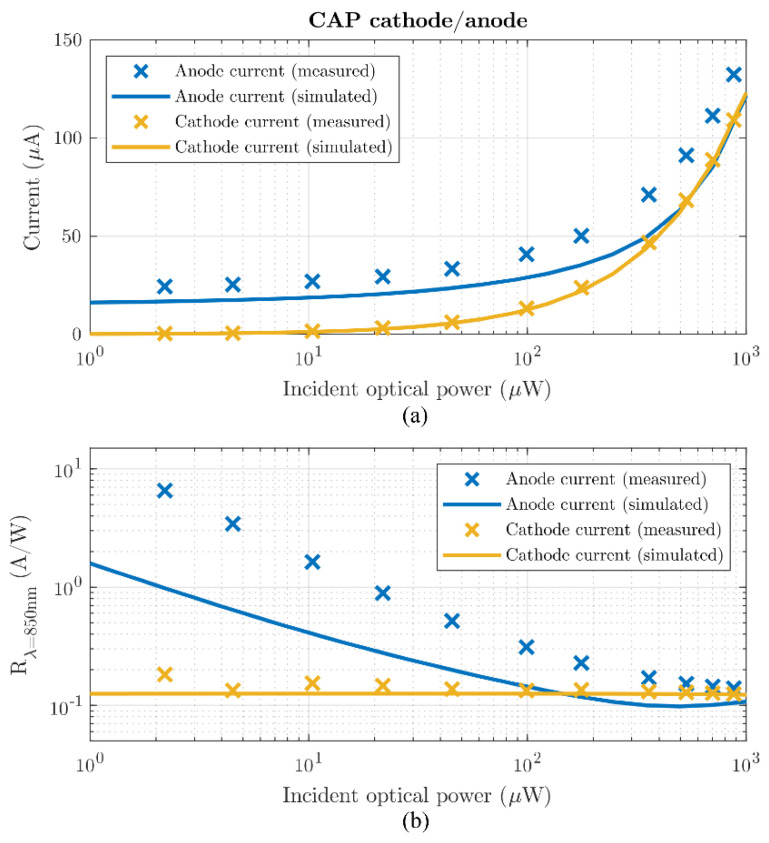
Measured and simulated anode and cathode current (**a**) and responsivity (**b**). V_anode_ = V_cathode_ = 0 V, V_ring_ = V_substrate_ = −5 V.

The measured responsivity shows that photo-gain is indeed feasible (shown in Figure 8). The cathode current responsivity is quasi constant over incident optical power, reinforcing the fact that all minority carriers are ultimately collected by the cathode n^+^ region. However, we see that the anode current responsivity also has a very significant contribution towards the total photocurrent, especially at low light intensities as supported by simulations.

The discrepancies between simulated and measured anode current can be traced to incomplete modeling of the junctions. Simulations show sensitivity to substrate doping levels, junction depths and surface trap state densities.

**Figure 9:**
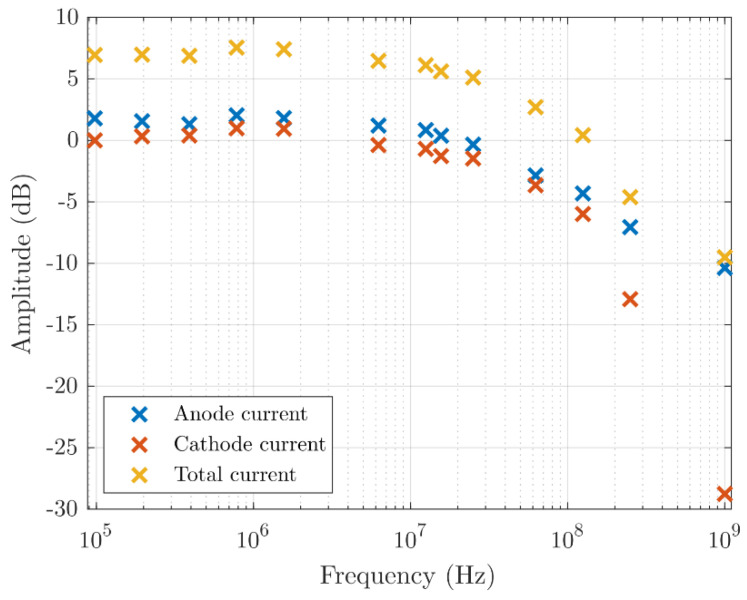
The normalized frequency response of the anode and cathode current at an average optical power of 360 µW, V_anode_ = V_cathode_ = 0 V, V_ring_ = V_substrate_ = −5 V.

The majority current contribution can also be significant at higher frequencies, as shown in Figure 9. It was measured by exciting an OPV300 VCSEL (Optek Technology, Carrollton, TX, USA) (λ = 850 nm) using a PRBS7 signal at multiple sampling frequencies. The sampled signal was analyzed at the excited frequency bins after windowing to reduce spectral leakage. The signals were measured using an Agilent Infinium DCA-J 86100C (Agilent Technologies, Santa Clara, CA, USA) with an 86117A 30 GHz channels module where the signals were terminated by a 50 Ω load resistance. The “true” optical input signal was simultaneously measured using a Thorlabs DXM12DF DC−12 GHz (Thorlabs, Inc., Newton, NJ, USA) detector using a beam splitter.

The measured frequency response shows that the anode current supports similar frequencies as the cathode current. To reach higher bandwidths with this type of detector, a viable option is to combine both the cathode and anode currents into one photocurrent. The result is a small photo-gain and an extended bandwidth through accumulation of minority carriers [30,31,32]. 

For noise, we can expect two dominant noise sources: shot noise is expected for minority current, while thermal noise is expected for the majority current. Since the minority current (and its shot noise) directly modulates the majority current (and so the generated thermal noise), a more involved approach is needed to model the overall noise behavior. Noise performance has not been measured and is subject to further research.

### 3.2. Current-Assisted Photonic Demodulator (CAPD)

The current-assistance principle allows to modulate the electrical drift field distribution through modulation of the voltage sources connected to the substrate ohmic contacts. This concept is employed in the current-assisted photonic demodulator (CAPD) devices that have been successfully used in numerous indirect TOF (iTOF) distance sensing applications.

**Figure 10:**
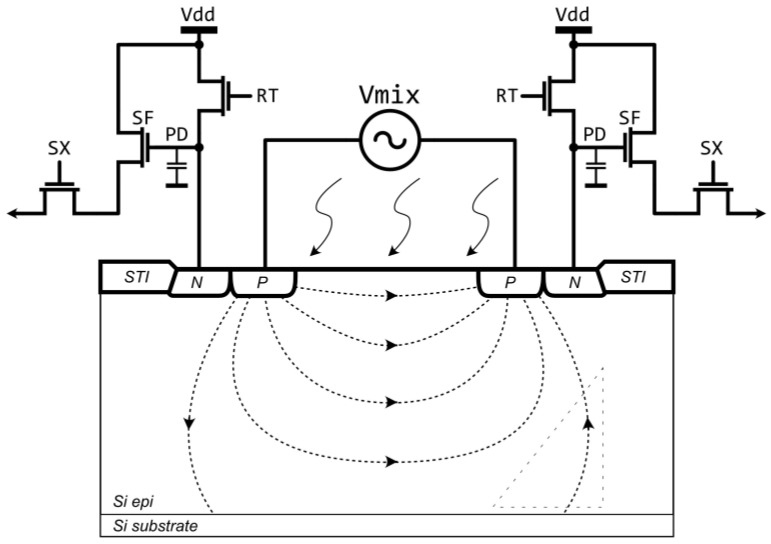
3-transistor (3T) 2 tap iTOF CMOS pixel architecture.

The CAPD device was described in detail in van Nieuwenhove et al. [12], and van der Tempel [33]. The general idea of the CAPD is illustrated in Figure 10. In this example, the photodetector is built in the lowly doped p-type substrate designated with a “Si epi” label. The CAPD is composed of a substrate resistor, formed between the two p^+^ regions (labelled “P”), and the two n^+^ regions (labelled “N”) located in the close proximity to the p^+^ regions. When a voltage source Vmix is connected to these p^+^ regions, a majority hole current will flow through the substrate resistor creating a gradual voltage drop and, therefore, an electric field in the silicon substrate. Light, incident on this device from the top, is absorbed in the substrate creating electron-hole pairs. The photo-electrons are drifting along the electric field lines towards the p region with a higher potential creating a pool of electrons around the p^+^-region. These photoelectrons cannot penetrate the p^+^-region because of a potential barrier stemming from the doping difference between the highly-doped p^+^-region and a lowly-doped p^-^-substrate. This electron pool is drained by placing an n^+^ doped region near p^+^-doped region. Therefore, the minority carrier electron current stemming from photo generation can be measured separately from the majority hole electrical current which assists in creating an electrical drift field in the substrate. Because of such separation, there is no extra thermal or shot noise contribution from the majority substrate current.

When an alternating voltage is applied to the p^+^-regions, the photo electron current can be guided either to one n^+^- region or the other, thus, an electronic shutter for the optical signal, controlled by the voltage source Vmix, is formed.

The photo-electrons accumulate in the n^+^ doped regions and the signal can be read out using circuits that are typically used in the conventional 3-transistor (3T) and 4-transistor (4T) image pixels. Figure 10 shows an example with 3T readout circuits connected to the 2 collecting n^+^ doped regions (taps). The operation of the readout circuits is similar to the conventional 2D image sensor readout. Both PD nodes are reset with reset RT transistors at the start of the integration, and the photo-electrons are integrated on the PD nodes which accumulate in-phase and out-of-phase photo-electron signals. At the end of the integration the voltage signals are read out through the source follower transistors SF and row select switches SX. The CAPD pixels can also have more complex readout circuitry like 4T readout circuits, sample and hold circuits for improved global shutter performance, circuits for dynamic range extension or common mode signal subtraction.

The iTOF principle is closely related to the lock-in signal detection technique developed in 1946 by R.H. Dicke to improve sensitivity of microwave radar receivers [34]. The lock-in technique allows signal recovery in noisy environments by advantageously exploiting prior knowledge about the signal itself. The lock-in correlation signal can have a higher signal to noise ratio (SNR) than the original signal since the signal is correlated to the reference signal, but the random noise is not, thus, enabling signal detection in noisy environments. CAPD devices can be used to measure the correlation signal between the optical signal and the reference electrical signal.

Although, an arbitrary electrical reference signal can be applied to the CAPD to modulate the drift field, a square wave signal is most often used in practice. A photonic demodulator can be modelled as an electronic shutter device which creates a temporal window ${\mathrm{rect}}_{\mathrm{CAPD}}\left(t\right)$ for the impinging optical signal what is equivalent to multiplication of the square wave reference signal and the impinging optical signal. The signal within this temporal window gets integrated on the sensor’s integration capacitor and the resulting voltage signal is then sampled. Mathematically this temporal windowing and integration processes can be described as a convolution or cross-correlation of the optical signal with a rectangular window function and sampling can be modelled by a Dirac comb function. Thus, the iTOF signal s(t) can be represented as:

(5) $$s\left(t\right)=\left[o\left(t\right){*\mathrm{rect}}_{\mathrm{CAPD}}\left(\frac{t}{t_{\mathrm{pd}}}\right)\right]\times \overset{+\infty }{\underset{n=-\infty }{\sum }}\delta \left(t-\frac{n\;T_{\mathrm{mod}}}{m}\right)$$

where o(t) is the incoming detected optical signal, $t_{\mathrm{pd}}$ is the temporal window width, $T_{\mathrm{mod}}$ is the modulation period, m is the number of phases, i.e., number of temporal windows per modulation period, and n is the integer sample number n∈ℤ . In a general case, at least three samples are required to recover the three unknown parameters in the incoming optical signal: amplitude, offset and phase shift. The most common iTOF sensor design with two photo-detector nodes (taps) creates two (in-phase and out-of-phase) temporal windows. However, such a two tap iTOF sensor can still be used to find the phase shift of the incoming optical signal: the necessary extra samples needed to find the three unknown parameters of the incoming light signal can be created by repeating the measurement with an extra phase shift introduced between the light signal and the CAPD electrical drift field modulation signal. The samples from multiple measurements may be combined producing a signal similar to the output signal from an iTOF sensor with a higher number of taps.

**Figure 11:**
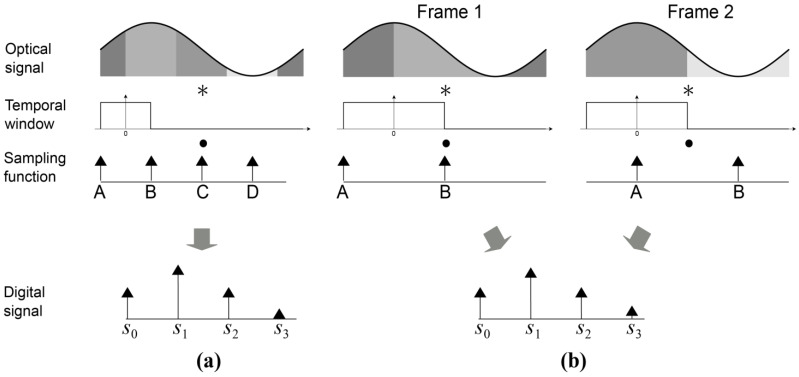
iTOF sampling for a 4 tap pixel (**a**) and a pseudo 4 tap pixel using 2 tap pixel (**b**). The * sign denotes the convolution in Equation (5).

Figure 11a illustrates the sampled TOF signal from a four tap iTOF sensor. Each tap A , B , C and D is sampling the signal integrated over its corresponding time window. These samples form a periodic time domain signal $s_{0},s_{1},s_{2},s_{3}$ .

Figure 11b illustrates a signal from two measurements using a two tap iTOF sensor. Taps A and B are sampling the signal integrated over its corresponding time window for two consecutive frame acquisitions, the frames having pi/2 phase shift. The four samples acquired over two frames can be recombined to form a periodic time domain signal similar to $s_{0},s_{1},s_{2},s_{3}$ .

In practice, the gating and integration process is repeated many times during the integration time $T_{\mathrm{int}}$ . For brevity, we will consider just one modulation cycle in the mathematical description of iTOF sensor operation.

After acquisition, the signal can be analyzed by means of a discrete Fourier transform (DFT). The first DFT complex valued bin contains information about the amplitude and the phase offset of the sampled iTOF signal’s first harmonic from which the TOF distance can be calculated.

For an m phase iTOF sensor, the DFT coefficients can be expressed as:

(6) $$S_{b}=\overset{m-1}{\underset{k=0}{\sum }}s_{k}\;e^{-{j\theta }_{k}\;b}\;=\;\overset{m-1}{\underset{k=0}{\sum }}s_{k}\;\left[\cos\left(\theta_{k}\;b\right)-j\;\sin\left(\theta_{k}\;b\right)\right],$$

where $\theta_{k}=2\pi \;\frac{k}{m}$ , and $s_{k}$ is the k -th iTOF sample. The DFT complex valued bins can be represented using the real and imaginary components $I_{b}$ and $Q_{b}$ :

(7) $$S_{b}=I_{b}\;-\;{\mathrm{jQ}}_{b}$$

where $I_{b}=\sum_{k=0}^{m-1}s_{k}\;\cos\left(\theta_{k}\;b\right)$ , and $Q_{b}=\sum_{k=0}^{m-1}s_{k}\;\sin\left(\theta_{k}\;b\right)$ . The TOF phase shift and amplitude signals can be then calculated using the first DFT bin as:

(8) $$\phi =\mathrm{atan}2\left(Q_{1},I_{1}\right)$$

(9) $$A=\sqrt{I_{1}^{2}+Q_{1}^{2}}$$

For example, for the case of 4 tap iTOF pixel ( m=4 ), the phase ϕ and amplitude A can be calculated as:

(10) $$I_{1}=s_{0}-s_{2},Q_{1}=s_{1}-s_{3}$$

(11) $$\phi =\mathrm{atan}2\left(s_{1}-s_{3},s_{0}-s_{2}\right),$$

(12) $$A=\sqrt{{\left(s_{0}-s_{2}\right)}^{2}+{\left(s_{1}-s_{3}\right)}^{2}}$$

The TOF distance to the object d is:

(13) $$d=\frac{\phi }{4\pi }\times \;c\;\times T_{\mathrm{mod}}$$

The second and higher order DFT bins can also be used to extract other useful iTOF signal indicators such as motion or saturation depth [35,36]. The iTOF distance error and SNR can be calculated as [6]:

(14) $$\sigma_{\mathrm{depth}}\equiv \frac{1}{\sqrt{2}\cdot \mathrm{SNR}}\times \frac{1}{2\pi }\times \frac{c}{2\cdot F_{\mathrm{mod}}}$$

(15) $$\mathrm{SNR}\equiv \frac{A_{\mathrm{pix}}\times \;R\;\times \;\mathrm{FF}\;\times {\;C}_{\mathrm{mod}}\times {\;\Phi }_{\mathrm{active}}\times {\;t}_{\mathrm{int}}\times \frac{1}{q}}{\sqrt{A_{\mathrm{pix}}\times \;R\;\times \;\mathrm{FF}\;\times \left(\Phi_{\mathrm{active}}+\Phi_{\mathrm{ambient}}\right)\times t_{\mathrm{int}}\times \frac{1}{q}+N_{\mathrm{system}}^{2}}}$$

where $A_{\mathrm{pix}}$ is the pixel area, FF —fill factor, R —responsivity, $C_{\mathrm{mod}}$ —demodulation contrast, $F_{\mathrm{mod}}$ —modulation frequency, $t_{\mathrm{int}}$ —integration time, $N_{\mathrm{system}}^{2}$ —variance of the system noise floor, $\Phi_{\mathrm{active}}$ is sensor irradiance due to the active illumination and $\Phi_{\mathrm{ambient}}$ is the sensor irradiance due to the ambient illumination.

To achieve the lowest distance noise, the product $\left[\sqrt{A_{\mathrm{pix}}\times \;R\;\times \;\mathrm{FF}}\right]\times \left[C_{\mathrm{mod}}\times {\;F}_{\mathrm{mod}}\right]$ should be maximized. The first factor $\left[\sqrt{A_{\mathrm{pix}}\times \;R\;\times \;\mathrm{FF}}\right]$ is defined by the optical to electrical signal conversion efficiency, and the second factor $\left[C_{\mathrm{mod}}\times {\;F}_{\mathrm{mod}}\right]$ is defined by the CAPD speed.

The current assistance principle allows to create electric drift field across large semiconductor volumes to achieve high $A_{\mathrm{pix}}$ , R and FF , thus maximizing $\left[\sqrt{A_{\mathrm{pix}}\times \;R\;\times \;\mathrm{FF}}\right]$ .

The CAPD speed factor $\left[C_{\mathrm{mod}}\times {\;F}_{\mathrm{mod}}\right]$ can be estimated from solution of the continuity equations [26] that govern the transport of the photo electrons:

(16) $$\frac{\partial n_{p}}{\partial t}=G_{n}-R_{n}+\nabla \times {\;J}_{n}+G_{L}$$

where $n_{p}$ is the minority carrier concentration in the p substrate, $G_{n}$ and $R_{n}$ are generation and recombination rates, $G_{L}$ is the generation rate due to impinging light signal, and $J_{n}$ is the electron current density:

(17) $$J_{n}={\mathrm{qn}\mu }_{n}E_{\mathrm{drift}}+{\mathrm{qD}}_{n}\nabla n$$

Detailed modelling of the photo currents is done in Estrada et al. [8]. An approximate estimation of the electron transient time across the length of the device can be done assuming linearly distributed electrical drift field across the device. For example, for a 15 µm device biased by a 1 V voltage source, its electron detection time can be approximately calculated as:

(18) $${\left[C_{\mathrm{mod}}\times {\;F}_{\mathrm{mod}}\right]}^{-1}\equiv \bar{t}\equiv \frac{\bar{d}}{\bar{E}\times {\;\mu }_{e}}=\frac{d^{2}}{V\;\times {\;\mu }_{e}}=\frac{15\times 15{\;µm}^{2}}{1V\;\times 1400{\;\mathrm{cm}}^{2\;}V^{-1\;}s^{-1}}\approx 1.6\;\mathrm{ns}$$

The above calculation gives a reasonable estimation of the average photoelectron detection time for an electron generated not far from the surface if the internal potential along the photoelectron travel paths towards n regions is monotonous and the electric field ( $V/\bar{d}$ ) is constant along the photoelectron travel path. However, in practice, this assumption does not always hold true.

Firstly, in case of NIR light, the optical signal can penetrate deep into the substrate where the electric field is weaker. To overcome this effect, CAPD devices that are designed for NIR wavelengths can have an extra negatively biased bottom electrode to create a vertical electric field component [37]. Alternatively, the backside illumination may be used to confine the generated photoelectrons to a smaller volume [6]: the light, that passed through the photosensitive silicon layer, is reflected from the metallization layers and then reenters the photosensitive volume effectively increasing the photosensitive silicon thickness.

**Figure 12:**
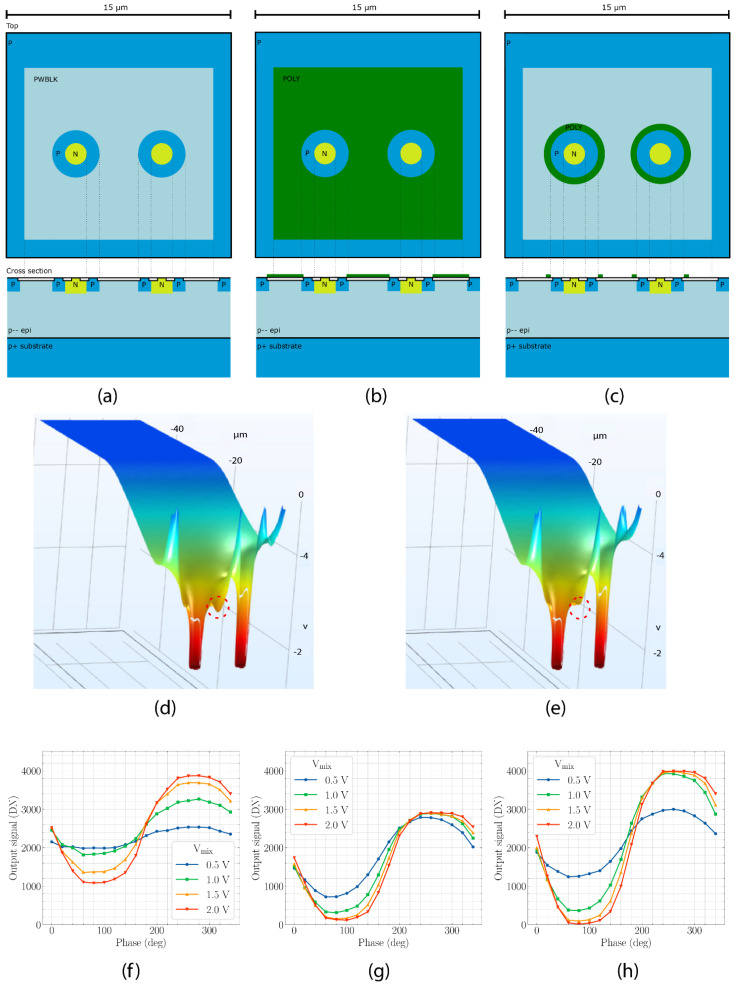
CAPD with a ring contact without a top polysilicon electrode (**a**), with a top polysilicon electrode (**b**), with a donut electrode (**c**,**d**) illustrates electrostatic potential under STI for device (**a**,**e**) illustrates electrostatic potential under STI for device (**b**,**c**) with a negative bias applied to the top electrode; (**f**–**h**) show measurement results of CAPD windowing function $rect_{\mathrm{CAPD}}$ for (**a**–**c**) respectively.

Secondly, when a modulation voltage source Vmix is connected to the biasing ohmic contacts (p^+^ regions) near the silicon surface, a slow drift region is formed under the ohmic contact with lower potential (illustrated with a dashed triangle in Figure 10) where the electric field is directed towards the substrate. Photo-electrons which are generated in this region are drifting towards a slow drift field region near the epi-substrate boundary and, when the polarity of the modulation voltage source Vmix is reversed, the photo-electrons are drifting towards the wrong detector n^+^-region lowering the demodulation performance. CAPD demodulation performance can be improved if the photogeneration under the ohmic contacts is prevented either by shielding, or by placing a microlens on top of the pixel focusing the light in the region with the strongest drift field ([6]), or by negatively biasing the substrate. Figure 12a–c [37] illustrates a CAPD architecture with a ring contact which is biased at a low potential and a modulation voltage Vmix is applied to the two ohmic contacts in the center. Such architecture reduces the mean travel path and the adverse effect of the slow region that may form under the negatively biased ohmic contact.

The assumption about monotonicity of the electrostatic potential may not hold in real devices leading to formation of charge pockets which deteriorate the device performance. Figure 12d illustrates the electrostatic potential under shallow trench isolation (STI). The potential around the p^+^-region, connected to the higher voltage, may have a local maximum due to the effect of the STI fixed positive charge. The Si/oxide interface has a positive surface-state charge which can vary in the order of $1\ldots 10^{11}\;Q/{\mathrm{cm}}^{2}$ depending on the technological conditions [38]. This positive charge bends energy bands forming charge pockets around the p^+^-region illustrated by the dashed red circle in Figure 12d. The charge pockets can trap photoelectrons leading to deterioration of the demodulation performance of CAPD sensor, as these trapped electrons can be detected by the wrong collector n^+^-region after the polarity of the modulation voltage source Vmix is reversed. The charge pockets around the p^+^-regions can be avoided by placing a negatively biased transparent electrode over the CAPD, as illustrated by Figure 12b, or by placing a donut shaped electrode around the p^+^-region where the charge pockets are formed [37], as illustrated by Figure 12c. The donut shaped electrode has an advantage over the solid electrode since reflection and absorption from polysilicon electrode does not lower the sensor responsivity as much, and the CAPD power consumption is not significantly increased because of formation of hole accumulation layer under the negatively biased electrode. Figure 12e illustrates the electrostatic potential under STI in the presence of the negatively biased electrode where the charge pocket was successfully removed. 

Figure 12f–h show measurement results of CAPD windowing function ${\mathrm{rect}}_{\mathrm{CAPD}}$ measured by illuminating the sensor with a narrow (~3 ns) optical pulse train signal and sweeping its phase while applying 50 MHz square-wave signals to the p^+^-regions. The characterized CAPD devices have 15 µm pitch and were manufactured in 180 nm technology. The CAPD without a transparent electrode over STI (f) shows poor demodulation performance due to charge pockets while having high signal amplitude (measured responsivity R=0.3 A/W ). The CAPD with a transparent polysilicon electrode shows near ideal demodulation; however, the signal swing is reduced (measured responsivity R=0.2 A/W ) compared to (f) due to reflection and absorption in the electrode. The CAPD device with the donut electrode (h) combines good demodulation performance with higher responsivity (measured responsivity R=0.3 A/W ).

#### Conclusions

The current-assistance principle allows to create strong electric drift fields in CAPD devices which are linearly distributed across large semiconductor volumes and which can penetrate deep in the semiconductor enabling high NIR sensitivity and cost-efficient manufacturing. CAPD devices are already widely used in numerous consumer and automotive applications. 

Table 1 compares different CAPD implementations and the Kinect2 photonic demodulator.

One of the further research frontiers for CAPD is extension of the spectral sensitivity range. The unique advantage of CAPDs over other common types of photonic demodulators is that the CAPD devices do not require high-quality oxides and can be manufactured in semiconductors like germanium which will allow sensing beyond the silicon sensitivity limit of ~1100 nm. Another potential research frontier is building CAPD sensors with a high number of taps that can be achieved, for example, by combining a gated demodulator with a current assistance principle [40].

### 3.3. Current-Assisted Photonic Sampler (CAPS)

The demodulation possibilities of current-assistance can also be optimized towards the creation of a sensor which operates as a fast shutter, i.e., in one state incoming light is detected with maximum efficiency and in a second state it is blind. Because photoelectrons generated in the second state do not need to be collected for readout but can just be disposed of, more compact and efficient structures can be employed for fast collection. 

This way, a gated sensor was created and called the current-assisted photonic sampler as it samples the light in a certain gate time-window. Operation is equivalent to that of an ICCD, but without the gain. The CAPS was developed for fast-gated detection of NIR fluorescence lifetime in which sub-nanosecond decay rates of fluorescent molecules are imaged. The requirement for very fast time-gate edges was the incentive to create a current-assisted sensor optimized just for gating. 

#### 3.3.1. Gated Detection of Fluorescence Lifetime

**Figure 13:**
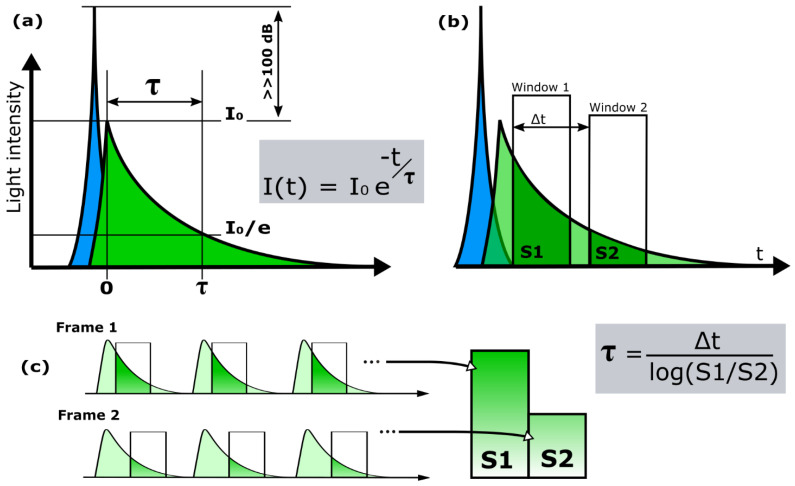
Fluorescence lifetime (τ) definition (**a**) 2-window gated lifetime measurement (**b**) and (practical implementation of the 2-window method for a gated camera (**c**).

The fluorescence lifetime (τ) is a parameter which describes the exponential decay rate of fluorescence after being excited with a pulse of light (Figure 13a). The fluorescence lifetime is the average time the molecule stays in an excited energy state after absorption of a photon and is an independent parameter which can be measured additionally to the spectral properties of fluorescence. The fluorescence lifetime depends on a number of things which each can be exploited for imaging:

1. The structure of the fluorescent molecule: imaging the lifetime can aid in revealing which fluorescent molecule is present or distinguish different fluorescent molecules [21].
2. The chemical environment of the fluorescent molecule: fluorescence lifetime has a sensitivity to chemical conditions such as pH or Ca+ or O_2_ concentrations and can as such be used as a probe for these conditions [41].
3. The distance to other fluorescent molecules: the fluorescence lifetime of a fluorescent molecule is influenced by another fluorescent molecule whose absorption spectrum overlaps with the emission spectrum of the first molecule and the influence is dependent on the proximity of the molecules. This property is exploited in a technique called time-domain FRET which is one of the most popular applications of fluorescence-lifetime imaging because the fluorescence-lifetime offers an absolute measure for the proximity in contrast to the relative intensities used in classic FRET [42].
4. The mobility of the fluorescent molecule: a molecule which is mechanically bound will lose less energy through non-radiative processes [43].

The fluorescence lifetime is typically in the nanosecond range and lifetimes as short as several hundreds of picoseconds are not an exception for NIR fluorescent molecules. This poses severe challenges for any system measuring and even imaging lifetimes. The gold standard in fluorescence-lifetime measurements is time-correlated single-photon counting (TCSPC) in which the arrival time of single photons is recorded and stored in a histogram which can then be analyzed with computationally expensive fitting methods. Implementing the electronics required for TCSPC in every pixel of an image sensor is difficult and TCSPC is usually either performed with a discrete single-photon detector for the analysis of a substance or with a scanning system for imaging in microscopy.

Fluorescence lifetime can also be detected with a gated detector in a family of methods which is called Rapid-Lifetime Determination (RLD). Figure 13b demonstrates the simplest incarnation of RLD in which the fluorescence intensity is measured in two equal time-windows at two different positions in the fluorescent decay separated by a time Δt. In the ideal case of a mono-exponential decay without background, the fluorescence lifetime can simply be calculated from Δt and the ratio of the signal detected in the two windows. The method can be extended to three windows to account for background signal and optimized window spacings and widths can be used for optimized lifetime measurement precision. Imaging fluorescence lifetime with an image sensor which implements a single gate-window can be performed by taking two frames at two different gate-window time positions. In each frame signal is collected from many consecutive fluorescence excitations. Finally, the lifetime is calculated from the integrated signal from frame 1 and frame 2 (Figure 13c).

#### 3.3.2. CAPS Operation Principle

The CAPS sensor has some features in common with the CAPD: the sensor is placed in a lowly-doped *p^-^*-substrate and around the sensor is a ring substrate contact at a negative potential, creating a substrate drift field that accelerates photo-generated electrons towards the center of the sensor.

**Figure 14:**
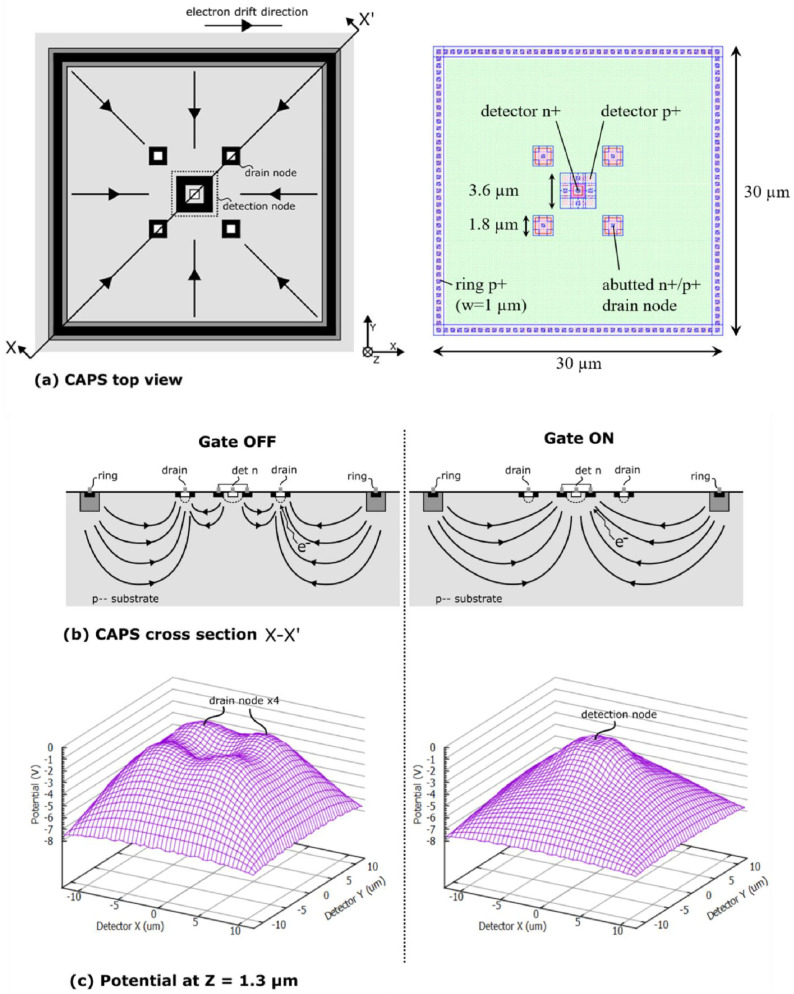
CAPS principle showing a top view of a CAPS sensor with detection node and four drain nodes with indication of drift field (**a**, left) and top view of the layout with dimensions indicated (**a**, right). CAPS cross section when the gate is OFF and when the gate is ON (**b**) and associated potential distributions at a depth of 1.3 µm (**c**).

The CAPS sensor consists of a centrally placed detection node surrounded with four drain nodes as shown in Figure 14a. The detection node consists of a n^+^ cathode surrounded by a p^+^ anode. Since the drain node is used to evacuate the carriers, a more compact structure is used, where n^+^ and p^+^ regions are abutted. Because the silicide layer provides conduction between the n^+^ and p^+^ region, a single contact can both drain away photo-generated electrons and apply the necessary hole currents for the generation of the drift field. As such, the p^+^ zone can be made narrower, resulting in a very short diffusion distance.

By setting the drain nodes to a higher potential than the detection node, the detection node becomes completely shielded from photo-generated electrons. In this situation the gate is said to be OFF. If instead the detection node is set to the highest potential, electrons will be allowed to flow into the detection node and the gate is turned ON [20]. 

Figure 14b,c show the gating effect in more detail. On the left, the complete shielding of the detection node can be seen when the gate is OFF. Electrons travel towards higher potentials and as such electrons from anywhere in the detector will end up at the drain nodes and not at the detection node. This results in very good gate OFF quality or high gating contrast (difference in detected signal between detector in ON and detector in OFF state). When the gate is ON, following the highest potential leads directly to the detection node and leads to high-speed detection of electrons.

This fast gating mechanism does not require the use of an image intensifier, avoiding the photocathode efficiency loss found in ICCD cameras. Furthermore, this gating mechanism requires modulation of only low voltages which can be handled by high-speed electronics at high repetition rates.

**Figure 15:**
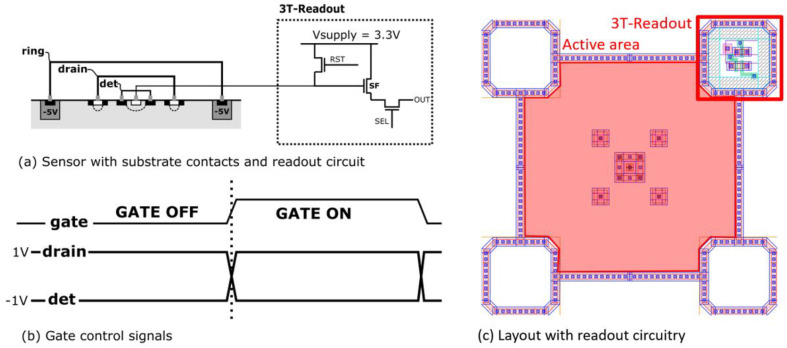
Typical operation of a CAPS sensor showing the sensor substrate contacts and 3T readout circuit (**a**) and gating control signals (**b**). The layout of the CAPS sensor with readout circuitry is shown in (**c**).

Figure 15 shows the typical potentials involved in the CAPS operation. The detection node is connected to a 3T-readout circuit for resetting of the detection node and buffering of the sense-node voltage by a source follower. At the start of every measurement, the sense node is reset by asserting the RST-signal. After having reset the sensor, the gate signal will be asserted at a fixed position in time with respect to the measured light and this for n cycles. In practice, this usually means that the gate is synchronized to the pulsed laser that excites the fluorescence and that the emission is gated for n repetitions of the laser pulse, during which the signal is integrated. The voltage on the sense node is then sampled by an ADC and stored as measurement result for the gate in position 1. After this first integration period, a new integration period starts with the gate in another position and the resulting voltage is sampled again. These two results can then be used to estimated fluorescence lifetime as described in Section 3.3.1 or the integration process can be repeated with the gate in more positions for extra measurement points.

#### 3.3.3. CAPS Sensor Characteristics

**Figure 16:**
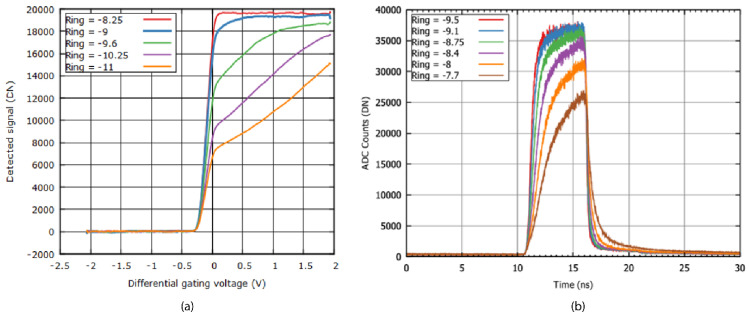
Gating behavior of the CAPS sensor in DC (**a**) and instrument response function (IRF) for a 4 ns gate (**b**).

Unlike the CAPD, the CAPS is not a symmetrical device. Due to its design with a centrally placed detection node surrounded by drains, it will be easier to direct electrons into the drains than into the detection node. This can be seen in the DC gating behavior, shown in Figure 16a: with increasing positive differential gating voltages, the photocurrent into the detection node rises more slowly than it decreases for negative voltages. The reason for the slower increase in detected electrons at positive gating voltages is the fact that electrons have to be directed past the drain nodes. It can also be seen that the ring voltage should not be too negative to achieve a high gating contrast and a sharp curve. The latter is important when the pixel is placed in an array: a voltage drop over the gating signal lines should not result in a different gating behavior.

Figure 16b shows a plot of the Instrument Response Function (IRF) for a 4 ns gating width at multiple ring voltages. This measurement allows to directly assess the lifetime measurement capabilities: the slope of the falling edge is used to determine the intrinsic decay time, while the rising edge determines the minimum gate window. It can be seen that at less negative ring voltages, the IRFs decay rate becomes slower, but also the IRF rises slower and at some point does not reach full amplitude. Therefore, more negative ring voltages are used for a higher QE and short gate widths. In practice, an optimum voltage is found where both a high gating contrast and high speed are achieved. 

#### 3.3.4. Fluorescence Lifetime Imaging

**Figure 17:**
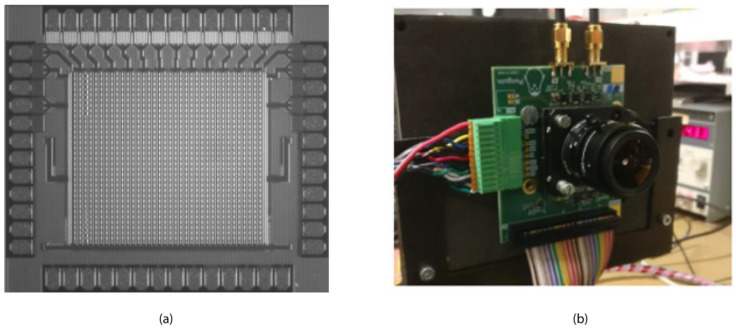
Micrograph of the 32 × 32-pixel CAPS array (**a**) and camera setup (**b**).

A first-generation 32 × 32-pixel CAPS array has been realized in a standard 350 nm CMOS process on a high resistivity (~1000 Ω·cm), thick p-type epilayer (14 µm). A camera is developed around this to demonstrate the capabilities of the CAPS-array [21]. The pixels in the array measure 30 × 30 µm with an active area of 480 µm^2^ resulting in a fill factor of 53%. The camera characteristics, listed in Table 2, are results for pixel [16,16] located in the center of the array. These characteristics are compared to commercially available Intensified CCD cameras in Table 3. A micrograph of the sensor die and the camera setup can be seen in Figure 17.

**Figure 18:**
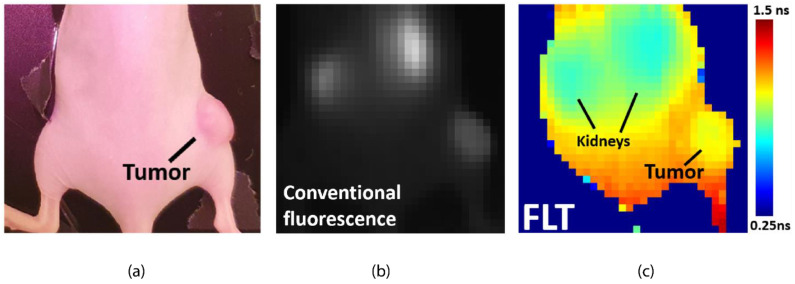
In vivo fluorescence experiment using a CAPS fluorescence-lifetime camera. A mouse bearing a xenograft tumor with EGFR expression and injected with an anti-EGFR nanobody-based NIR fluorescence contrast agent (**a**). Both tumor and kidneys light up under conventional fluorescence imaging (**b**). CAPS fluorescence-lifetime imaging reveals a fluorescence lifetime difference between the tumor and kidneys (EMIM 2020) (**c**).

Practical use of this 32 × 32 CAPS image sensor has been demonstrated in a proof-of-concept for fluorescence-guided surgery [18]. Fluorescence contrast is increasingly used in surgical guidance to light up blood flow, tumors, nerves, and more. Because of its ability to penetrate a few millimeters of tissue, the wavelength of preference is around 800 nm. This leaves little spectral room to image more than one contrast agent based on spectral differences. In the proof-of-concept, the CAPS image sensor is used for fluorescence-lifetime imaging of an ex-vivo mouse phantom with two 800 nm fluorescence inclusions of dyes (ICG and IRDye-800CW) but with a different fluorescence lifetime [18]. The CAPS camera is able to distinguish both dyes based on a difference in fluorescence lifetime whereas a conventional fluorescence camera cannot. More recently, at the EMIM 2020 conference on molecular imaging, the same camera has been demonstrated in an in vivo experiment. In the experiment, a tumor-bearing mouse was injected with an anti-EGFR nanobody-based fluorescence contrast agent. When imaged in conventional fluorescence mode the tumor lights up. However also the kidneys light up. Only in the CAPS fluorescence-lifetime image can the difference between kidneys and tumor be seen, demonstrating that fluorescence-lifetime can be used as modality to distinguish Fluorescence emission from the targeted tissue (tumor) and non-specific emission (in this case kidneys) (Figure 18).

#### 3.3.5. Conclusions

By combining good NIR quantum efficiency with fast gating, the CAPS image sensor seems a good candidate for in vivo fluorescence imaging in which NIR dyes play a crucial role and in which a fluorescence-lifetime imaging capability can enable new possibilities for future applications such as the imaging of fluorescence contrast agents simultaneously in complex surgical procedures or increasing the contrast specificity by taking fluorescence lifetime contrast into account. 

### 3.4. Current-Assisted SPAD (CA-SPAD)

Single-photon avalanche diodes (SPADs) are widely popular due to their single-photon sensitivity and CMOS compatibility [47,48,49,50,51,52,53]. CMOS compatibility and inherent digital nature of the SPAD detection leads to quenching and timing processing circuitry to be constructed around the SPAD pixel. A recent work presenting the realization of a Megapixel SPAD imager further demonstrates the advancements made in the field of SPADs fabricated in CMOS technologies [47]. Frontside-illuminated (FSI) SPADs have limited NIR sensitivity, often because the device is enclosed in a deep N-well, in order to isolate the moderately high voltages of the SPAD. As seen in Section 2, increasing the absorption layer thickness alone does not lead to an increase in photo-sensitivity. Therefore, an idea of a novel SPAD detector with a small p-n junction and a large photo-absorption volume with drift field was conceived. The drift field is due to the potential gradient in the absorption volume, arising from applying a bias between two terminals. In short, we integrate the current-assistance principle with a small junction SPAD to increase its photo-absorption volume with swift response speed. To stick with the convention, this novel SPAD is called “current-assisted SPAD (CA-SPAD)”.

**Figure 19:**
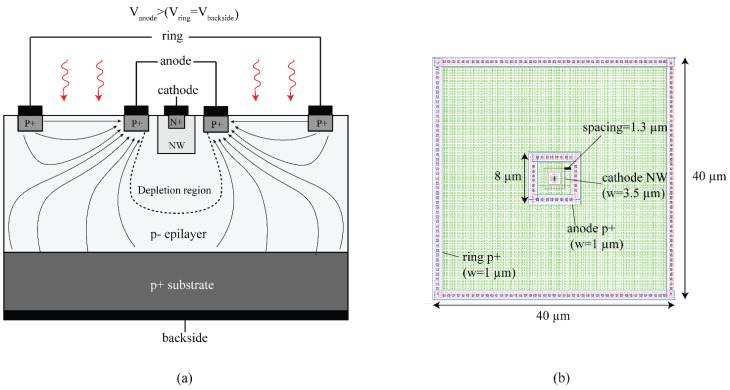
Cross section of CA-SPAD-1, lines with arrows indicate the direction of photo-electrons, dotted line represents the boundary of the depletion region (**a**) and top-view of the layout of CA-SPAD-1 with dimensions indicated (**b**).

The first proof-of-concept device (we will refer to it as CA-SPAD-1 [24]) was fabricated in X-fab foundry’s 350 nm CMOS (XO035). Mask layers readily available for standard transistor formation were used and no special layers were used. Hence, the fabrication is done in a relatively low-cost and accessible CMOS technology. This technology has a high resistivity (~1000 Ω·cm), thick p-type epilayer (14 µm) which enables possibility of good NIR sensitivity. The illustrated cross-section is shown in Figure 19a. The central SPAD is comprised of a N-well cathode surrounded by a p^+^ anode. N-well is chosen covering the n^+^ doping, to form the cathode (detect node in other current-assisted detectors), to reduce band-to-band tunneling at large reverse bias voltages. A consequence of having an N-well is the increased breakdown voltage. This central p-n junction is surrounded by a much larger p^+^ “ring”, which marks the boundary of the photo-detection area. A potential difference is applied between the anode and the ring, which results in drift field between them. The anode is biased more positively with respect to the ring in order to attract the photo-generated electrons towards the anode. The photoelectrons which reach the anode will have to only diffuse a small distance before falling into the depletion region and commencing an avalanche breakdown. There is a steady hole current between the anode and the ring, due to the potential difference, leading to a steady power dissipation per pixel which is a small price to pay to enable a high-speed operation. 

**Figure 20:**
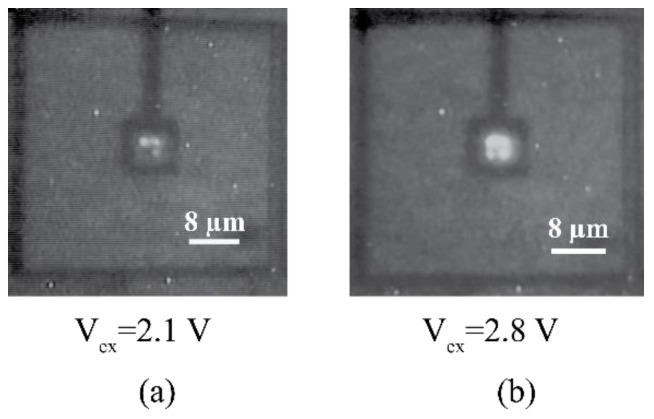
Light emission test to demonstrate reverse breakdown occurring at sharp corners at V_ex_= 2.1 V (**a**) and V_ex_= 2.8 V (**b**).

CA-SPAD-1 had square structures which are spaced adequately (shown in Figure 19b) to comply with the design rules with some margin. Passive quenching (external to the chip) was used to characterize the device behavior and a few important issues were identified. The primary issue was the sharp corners created by the square geometry. Sharp corners are known to concentrate the electric field which lead to selective breakdown [54,55,56]. This resulted in limited photon detection probability (PDP) and increased timing jitter due to the non-uniform electric field. Light emission tests show discrete light emission spots from sharp corners, further suggesting that the breakdown occurs primarily at the sharp corners (shown in Figure 20). The secondary issue was the lack of on-chip quenching circuitry which resulted in large deadtimes (~50 ns) due to increased parasitic capacitance from the bondpads (~1 pF). While the SPAD p-n junction capacitance is estimated to be ~1 fF, large parasitic capacitance also means that there is more current required to quench the avalanche operation. This results in more carriers flowing through the p-n junction during an avalanche event which increases the after pulsing probability (APP). Although the device did not perform up to the existing FSI SPADs, it was vital in demonstrating that the principle works as expected and the main issues were identified.

**Figure 21:**
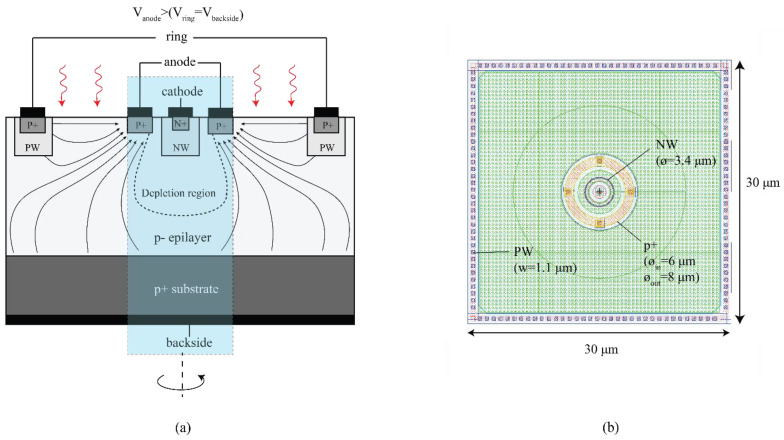
Cross section of CA-SPAD-2, lines with arrows indicate the direction of photo-electrons, the dotted line represents the boundary of the depletion region (**a**) and top-view of the layout of CA-SPAD-2 with dimensions indicated (**b**).

The second iteration (we will refer to it as CA-SPAD-2 [25]) was also fabricated in X-Fab’s XO035 technology. A few changes were made in the design of the pixel: (1) The junction was made with a cylindrical symmetry to avoid sharp corners (shown in Figure 21), (2) The “ring” terminal is chosen to be a P-well (with a p^+^ contact) to realize a more ohmic contact, (3) The pixel pitch was reduced to 30 × 30 µm. An on-chip passive quenching resistor (100 kΩ) was used for quenching. 

**Figure 22:**
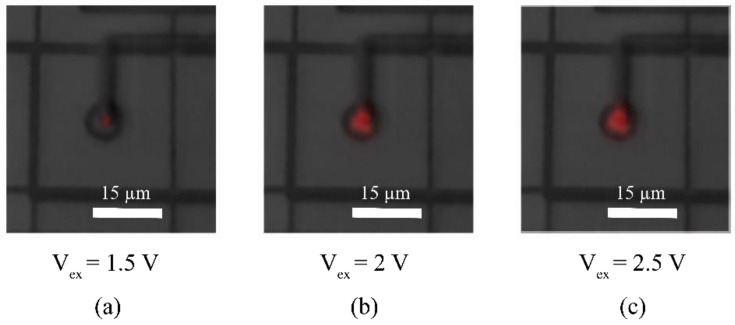
Light emission from “central SPAD” area at V_ex_= 1.5 V (**a**)**,** V_ex_= 2 V (**b**) and V_ex_= 2.5 V (**c**). The light emission is false-colored red.

**Figure 23:**
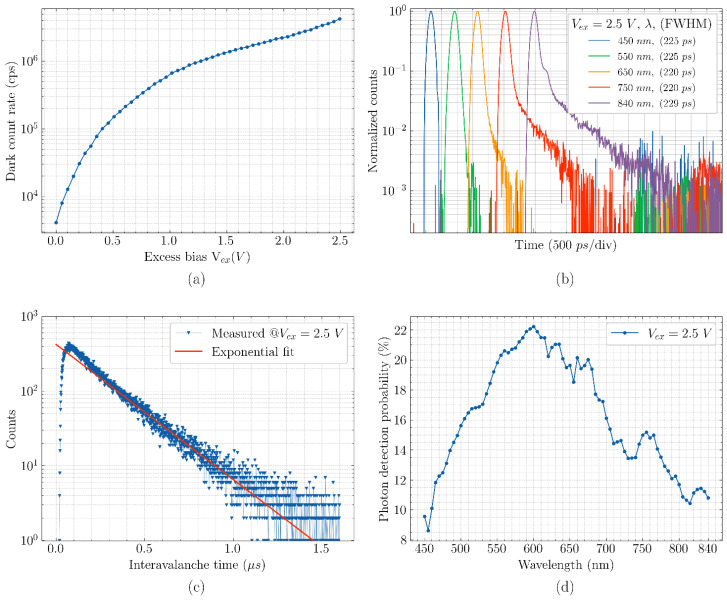
Dark count rate as a function of excess bias voltage for CA-SPAD-2 (**a**), Timing response for different wavelengths for CA-SPAD-2 (**b**), Inter-avalanche histogram to characterize after-pulsing probability for CA-SPAD-2 (**c**), and photon detection probability (PDP) as a function of wavelength for CA-SPAD-2 (**d**).

A light-emission test for CA-SPAD-2, shown in Figure 22, shows one uniform light emission spot suggesting that breakdown is uniform in the p-n junction. This is also confirmed by the improved timing precision shown in Figure 23b and photo detection probability shown in Figure 23d. Dark count rate is still large, shown in Figure 23a and is primarily due to the N-well/p^+^ junction construction. Afterpulsing probability at V_ex_ of 2.5 V is 13% which could possibly be reduced by efficient quenching circuits.

A main concern influencing CA-SPAD-2 performance parameters, such as afterpulsing and PDP, is the ineffective quenching. At larger excess bias voltages, the deadtime was much larger. This could be due to the small junction capacitance of the CA-SPAD-2 and re-triggering of the SPAD by carriers, generated by an avalanche event, due to fast recharge. This effect is further explained in Jegannathan et al. [25]. This “re-triggering” leads to extended deadtimes which could saturate the detector at high counting rates. An effective active quench and recharge circuit would possibly eliminate this effect and the performance can be further improved.

The performance of the CA-SPADs can be further improved when having the control over the device process (such as doping levels, thickness). Compared to conventional SPADs, CA-SPADs can offer benefits such as a scalable pixel pitch that has an absorption region decoupled from the avalanching region, a low-capacitance p-n junction and swift and efficient photo-response for NIR wavelengths. The two major challenges with the CA-SPADs fabricated in the 350 nm CMOS technology include high breakdown voltage which makes it difficult to place circuitry close by and a high dark count rate due to the topology of the p-n junction. Both these challenges can be addressed when going to a back-side illuminated CA-SPAD pixel engineered with custom doping layers. The performance parameters of the two CA-SPAD iterations are compared in Table 4.

## 4. Conclusions

The use of current assistance in several types of photodetectors for different applications has been reviewed (overview in Table 5). Most of the given examples were based on available drain-diffusions, N&Pwells and epilayer that are available in standard 350 nm CMOS for making NMOS and PMOS transistors. Advanced technology options available in finer feature-size CMOS could further enhance the detection/demodulation/sampling specifications. Custom doping profiles could also lead to far better field application topologies and improve operation even further, however at the cost of additional investment. 

## Figures and Tables

**Table 1:** CAPD performance parameters.

Parameter	MLX75023	MLX75024 (Donut Variant)	Kato et al. [39]	Kinect2
Tech. node	0.35 µm	0.18 µm	90 nm	0.13 µm
Pixel pitch	15 µm	15 µm	10 µm	10 µm
$C_{\mathrm{mod}}@50\;\mathrm{MHz}$	60%	85%	91%	68%
R @ 850 nm	0.2 A/W	0.3 A/W	0.34 A/W	-
Pixel FF	35% (native)	70% (native)	>80% (µlens)	60% (µlens)

**Table 2:** Proof-of-concept CAPS camera characteristics.

Resolution	32 × 32
Pixel size	30 × 30 µm
Fill factor	53%
External (effective) quantum efficiency @780 nm	25%
Internal (effective) quantum efficiency @780 nm	71%
Minimum gate width	500 ps
Maximum gate width	Up to the laser repetition period
Gate position time resolution	11 ps
Gate position range	Full laser repetition range
Maximum gate repetition rate	>100 MHz

**Table 3:** CAPS proof-of-concept camera compared to commercially available Intensified CCD cameras.

Camera	Photocathode	NIR QE @ 780 nm	Minimum Gate Window	Maximum Gate Repetition Rate
La Vision PicoStar HR [44]	Gen II	<8%	300 ps	110 MHz
Andor iStar U [45]	Gen II	<10%	2 ns	500 kHz
Andor iStar U [45]	Gen III	<24%	2 ns	500 kHz
Princeton Instruments PI-MAX4 [46]	Gen II	<12%	500 ps	100 kHz
Princeton Instruments PI-MAX4 [46]	Gen III	<27%	500 ps	100 kHz
CAPS camera today	NA	<25%	500 ps	>100 MHz
CAPS camera possibility	NA	<71%	500 ps	>100 MHz

**Table 4:** Performance parameters of CA-SPADs.

Device	CA-SPAD-1 [24]	CA-SPAD-2 [25]
CMOS process	350 nm	350 nm
Foundry, Technology	X-Fab, XO035	X-Fab, XO035
Pixel pitch, shape	40 µm, square	30 µm, square
Junction, shape	N-well/p- epi/p^+^, square	N-well/p- epi/p^+^, cylindrical
Breakdown voltage	51 V	48 V
Excess bias voltage V_ex_	0.86 V	2.5 V
Timing jitter	370 ps	220 ps
PDP @ 785 nm [V_ex_]	5.6% [1.1 V]	11.6% [2.5 V]
Afterpusling probability	5.7%	13%

**Table 5:** Summary of current-assisted photodetectors.

Device	Year	Number of Pixels (Recent)	Applications
CAPD	2005	320 × 240 [39]	Indirect time-of-flight imaging
CAPS	2015	32 × 32 [21]	Fast time-gated imaging, fluorescence lifetime imaging (FLI)
CAP	2019	1 [23]	Optical receivers
CA-SPAD	2019	1 [25]	Direct time-of-flight imaging, low-light imaging, FLI

## Data Availability

The data presented in this study are available on request from the corresponding author.
