# Correction: Jegannathan et al. An Overview of CMOS Photodetectors Utilizing Current-Assistance for Swift and Efficient Photo-Carrier Detection. *Sensors* 2021, *21*, 4576

**DOI:** 10.3390/s22072610

**Published:** 2022-03-29

**Authors:** Gobinath Jegannathan, Volodymyr Seliuchenko, Thomas Van den Dries, Thomas Lapauw, Sven Boulanger, Hans Ingelberts, Maarten Kuijk

**Affiliations:** 1 Department of Electronics and Informatics (ETRO), Vrije Universiteit Brussel, 1050 Brussels, Belgium; vse@melexis.com (V.S.); Thomas.Van.Den.Dries@vub.be (T.V.d.D.); Thomas.Lapauw@vub.be (T.L.); sven.boulanger@vub.be (S.B.); hans.ingelberts@vub.be (H.I.); mkuijk@vub.be (M.K.); 2 Melexis, Transportstraat 1, 3980 Tessenderlo, Belgium

There was an error in the original publication, in Equation (5). The sign × needs to be changed to ∗ (the sign for convolution). In Section 3.2, Equation (5) appears as:

(5) $$s\left(t\right)=\left[o\left(t\right){\;\times \;\mathrm{rect}}_{\mathrm{CAPD}}\left(\frac{t}{t_{\mathrm{pd}}}\right)\right]\times \sum_{n=-\infty }^{+\infty }\delta \left(t-\frac{n\;T_{\mathrm{mod}}}{m}\right)$$

and it should be changed to:

(5) $$s\left(t\right)=\left[o\left(t\right){\;*\;\mathrm{rect}}_{\mathrm{CAPD}}\left(\frac{t}{t_{\mathrm{pd}}}\right)\right]\times \sum_{n=-\infty }^{+\infty }\delta \left(t-\frac{n\;T_{\mathrm{mod}}}{m}\right)$$

The original publication has also been updated [1].
